# CD105 (Endoglin) exerts prognostic effects via its role in the microvascular niche of paediatric high grade glioma

**DOI:** 10.1007/s00401-012-0952-1

**Published:** 2012-02-07

**Authors:** Stuart J. Smith, Hanna Tilly, Jennifer H. Ward, Donald C. Macarthur, James Lowe, Beth Coyle, Richard G. Grundy

**Affiliations:** 1 Children’s Brain Tumour Research Centre, Medical School, Queen’s Medical Centre Campus, University of Nottingham, Nottingham, UK; 2 Department of Neurosurgery, Nottingham University Hospitals NHS Trust, Nottingham, UK; 3 Department of Neuropathology, Nottingham University Hospitals NHS Trust, Nottingham, UK

**Keywords:** Glioblastoma, Angiogenesis, Endoglin, CD133

## Abstract

Paediatric high grade glioma (pHGG) (World Health Organisation astrocytoma grades III and IV) remains poor prognosis tumours, with a median survival of only 15 months following diagnosis. Current investigation of anti-angiogenic strategies has focused on adult glioblastoma multiforme (GBM) with phase III trials targeting vascular endothelial growth factor continuing. In this study we investigated whether the degree of vascularity correlated with prognosis in a large cohort of pHGG (*n* = 150) and whether different vessel markers carried different prognostic value. We found that CD105 (endoglin) had a strongly significant association with poor prognosis on multivariate analysis (*p* = <0.001). Supervised hierarchical clustering of genome wide gene expression data identified 13 genes associated with differential degrees of vascularity in the cohort. The novel angiogenesis-associated genes identified in this analysis (including *MIPOL*-*1* and *ENPP5*) were validated by realtime polymerase chain reaction. We also demonstrate that CD105 positive blood vessels associate with CD133 positive tumour cells and that a proportion of CD105 positive vessel cells demonstrates co-positivity for CD133, suggesting that the recently described phenomenon of vasculogenic mimicry occurs in pHGG. Together, the data suggest that targeting angiogenesis, and in particular CD105, is a valid therapeutic strategy for pHGG.

**Electronic supplementary material:**

The online version of this article (doi:10.1007/s00401-012-0952-1) contains supplementary material, which is available to authorized users.

## Introduction

In children high grade gliomas (HGG) (WHO astrocytoma grade III and IV) represent around 10% of intracranial neoplasms [14] and continue to have poor median survival rates of 14 [43] to 18[4] months. Brain tumours are the leading cause of cancer-related death in children, accounting for 10,000 lost years of expected life per year in the UK. Current multi-modal therapy of attempted gross total surgical resection, followed by radical radiotherapy and cytotoxic chemotherapy, can cause substantial morbidities with limited survival benefits. HGG are highly vascular tumours suggesting a potential novel therapeutic approach to target angiogenesis, thereby controlling tumour growth by disrupting the vascular network that supplies and supports this process [37].

Current phase III trials in adult GBM are targeting the pro-angiogenic ligand vascular endothelial growth factor (VEGF) via monoclonal antibody or small molecule inhibitor therapy [1]. Recent studies by our group and others have demonstrated substantial molecular differences between adult and paediatric HGG [35]. It is therefore important to quantify whether angiogenesis has as critical a role in childhood tumours as in adults and whether variant molecular pathways control the process.

Glioma vasculature formation occurs through at least three distinct processes. Angiogenesis is the process of generating new blood vessels from rerouting and remodelling of pre-existing vessels [37]. Vasculogenesis (blood vessel arrangement) was classically considered as an embryonic process, but has since been identified in tumours as the de novo formation of primitive blood vessels by the differentiation of circulating bone marrow-derived endothelial progenitor cells [40]. In addition, the process of vasculogenic mimicry [39] provides a contribution to tumour vasculature [45] by trans-differentiation of glioma cells into tumour-derived endothelial cells [42].

Among solid tumours, glioblastoma multiforme displays the most angiogenic features and the highest degree of vascular proliferation and endothelial cell hyperplasia [7]. Malignant gliomas require angiogenesis to establish a source of nutrients and oxygen and to eliminate cellular waste products [16]. Studies have also established that the tumour vasculature creates a protective ‘microvascular niche’, within which tumour-initiating cells can resist therapy [8]. Angiogenesis is thus a key pathologic event in HGG and necessary for the progression of a localised neoplasm to a highly aggressive tumour. The molecular processes involved in the ‘angiogenic switch’ are still being elucidated, with it remaining unclear whether the same factors predominate in children as in adults.

Conventional immunohistochemical measurement of microvessel density as a proxy marker for angiogenic activity has utilised markers of mature endothelial cells such as CD31 (PECAM), CD34 or factor VIII. Variable results have been achieved, both in HGG [28] and other tumour types [38] with some groups finding strong correlations with prognosis, but other centres finding no link to outcome [6].

CD105 (cluster of differentiation molecule 105) (endoglin) is a transmembrane homodimer protein of 180 kDa which is characteristically found on endothelial (blood vessel) walls [17]. It is a key component of the transforming growth factor β (TGFβ) receptor signalling pathway, involved with both receptors 1 and 2 [15]. CD105 fulfils key roles in angiogenesis and vasculogenesis during development (possibly through preventing apoptosis in hypoxic endothelial cells [29]) and mice that are null for the CD105 allele die due to aberrant yolk sac and cardiac development. The autosomal dominant condition hereditary haemorrhagic telangiectasia (Osler-Weber-Rendu syndrome type 1) is associated with mutations in the CD105 gene (located on chromosome 9) and thalidomide has been proposed for this condition as a way of stimulating vessel maturation [27].

As a marker for blood vessels CD105 is associated with immature vessels, and some studies have suggested it could be a marker that preferentially stains for novel angiogenic vessels. It has also been proposed that CD105 is a marker for mesenchymal stem cells [9]. In some tumour types it has been correlated negatively with prognosis [50], but its role in HGG (particularly in childhood) is not yet clear [2, 6, 19, 34, 47]. Because of its apparent specificity for tumour-associated blood vessels, CD105 is also of interest as a therapeutic target, with monoclonal antibody therapy in early stage clinical trials [18].

Because of its association with developing blood vessels, we hypothesised that CD105 may be a more representative measure of angiogenic activity in pHGG tissue, and that genetically characterising tumours with differing levels of CD105 may identify novel genes involved in paediatric angiogenesis. The micro-environment in immediate proximity to blood vessels has been suggested as an area enriched for, and supportive of, tumour initiating cells [10], such as those identified by CD133 positivity. We therefore also postulated that, as newly generated vasculature, CD105 positive vessels may be especially crucial to this microvascular niche, and may even be in part the product of tumour initiating cells (the process of vasculogenic mimicry). Overall, we demonstrate that CD105 is a superior prognostic marker compared to other vasculature-associated molecules in pHGG, that novel genes are associated with levels of angiogenic activity in pHGG and that CD105 positive vessels associate with CD133 positive tumour cells.

## Materials and methods

### Tumour samples

The tissue microarrays utilised in this paper were built from 150 samples banked by the Children’s Cancer Leukaemia Group (UK CCLG) through Nottingham Children’s Brain Tumour Research Centre (www.CBTRC.org). All samples were surgically collected ante-mortem at UK paediatric neurosurgical centres and are from paediatric high grade gliomas (both supratentorial and brainstem) with diagnosis confirmed by central pathological review (JL and Keith Robson). The cohort consisted of 102 children diagnosed with GBM, 29 with anaplastic astrocytoma (AA), 10 with HGG not otherwise specified, 8 with anaplastic oligodendroglioma and 1 with anaplastic pleomorphic xanthoastrocytoma. Full consent and ethical approval has been obtained for their use in this study, from the UK Children’s Cancer and Leukaemia Group and local ethical and Trent MREC approval (06/MRE04/86). A full clinical dataset, including demographic and treatment details, is available for each of the samples in this cohort. Three cores from representative regions of each tumour were included on the tissue microarray.

The gene expression analysis utilised a cohort of 53 de novo pHGG analysed by our group and collaborators as detailed previously [35], with the array data publicly available (GEO accession number GSE19578). All these samples also underwent central pathological review. Sixteen of these samples subjected to gene expression analysis were included on the tissue microarrays.

### Immunohistochemistry

Immunohistochemistry against the tissue microarrays commenced with de-paraffinisation in ethanol and xylene, followed by antigen retrieval in sodium citrate in a steamer for 40 min (for Ki67, CD31, CD133 and VEGF). For CD105, a proteinase K antigen retrieval method was utilised. After deparaffinisation, sections were covered with 100 μl of proteinase K solution (Sigma-Aldrich UK) (20 μg/ml). Specimens were then placed in a humidification chamber and then warmed to 37°C for 15 min in an oven. Anti-KI 67 (Dako monoclonal mouse anti-human Ki 67 antigen, clone MiB-1), anti-CD31 (Dako monoclonal mouse anti-human CD31, endothelial cell, clone JC70A), anti-CD105 (Dako monoclonal mouse anti-endoglin, SN6 h), and anti-VEGF (Dako monoclonal mouse anti-human vascular endothelial growth factor*,* Clone VG1) were all utilised at 1:50 concentration. Anti-KI 67 was applied for 30 min, all other antibodies were applied for 1 h at room temperature. Secondary antibody (100 μl Dako horse radish peroxidase conjugated rabbit anti-mouse) was applied for 30 min at room temperature. Finally, 2 μl of 3,3′-Diaminobenzidine chromogen in 98 μl of Dako REAL substrate buffer was applied before haemotoxylin counterstaining and mounting. At least three TMA cores were analysed for each tumour for each antibody, with the area of strongest staining in each core being assessed. For Ki67, scoring was performed by taking the most positive high powered field for each core and counting the number of positive nuclei as a percentage of the total number of nuclei present. When assessing Ki67 staining in vessel wall cells, all the blood vessels definitively identifiable as such in the core were first selected. The number of cell nuclei in the vessel wall staining positive for Ki67 were then counted as a percentage of the total number of nuclei in the vessel wall. Mitotic index was calculated by counting the number of mitotic figures per high powered field for at least three representative fields for each tumour. For CD31 and CD105, the total number of positive vessels per TMA core were counted. On whole sections of 12 paediatric HGG, defined areas of geographic necrosis were selected and the distance to the 10 nearest blood vessels staining positive for CD31 or CD105 was measured. For VEGF, as a cytoplasmic stain, scoring was undertaken by assessing the approximate percentage of cells staining positive (− = 0–1%, + = 1–5%, ++ = 5–20%, +++ = >20%).

### Immunofluorescence

Deparaffinisation was undertaken with ethanol and xylene washes, followed by antigen retrieval in 1 mM ethylenediaminetetraacetic acid (EDTA) buffer, adjusted to pH 8.0, heated in a steamer for 40 min. Blocking solution was applied (10% normal goat serum (NGS), 0.1% Triton X-100 in PBS, 1% bovine serum albumin) for 1 h in the dark at room temperature. Primary antibodies (CD31 and CD105 as above; CD133 Abcam rabbit polyclonal) were applied overnight in the dark at 4°C. Secondary antibody combinations of Alexa488-conjugated goat anti-rabbit (1:200) and Alexa555-conjugated goat anti-mouse (1:200), diluted in 2% NGS antibody diluent were then applied for 2 h in the dark at room temperature. Vectashield with DAPI mounting medium (CA94010, Vector Laboratories, Peterborough) was used. Images were taken using a Nikon ECLIPSE 90i light microscope fitted with a Hamamatsu OCRA-ER camera, using three fluorescent light filters; DAPI (excitation 340–380 nm), FITC (Ex 405–495 nm) and Texas-red (Ex 540–580 nm) and Volocity 5.0 imaging software. On average three images were taken per core wherever positive staining for CD31 or CD105 blood vessels was visible. Measurements were performed using the line measurement tool in Volocity; the distance between the centres of the nuclei for CD133+ cells was measured to the edge of the closest CD31+ or CD105+ blood vessel (see online resource 1). This was also performed for all CD133− cells in each image.

### Gene expression validation by quantitative real-time polymerase chain reaction

As previously detailed [35], analysis was conducted using the Affymetrix U133 plus2 platform and array data have been deposited at the Gene Expression Omnibus Web site (http://www.ncbi.nlm.nih.gov/geo/, accession No.GSE19578).

RNA was isolated from representative tumour specimens using the mirVana RNA isolation kit (Ambion, Austin, Tx). cDNA was produced with the RT^2^ First Strand Kit (Qiagen). Target genes identified on array analysis were validated with quantitative real-time PCR using a CFX96 realtime system (BioRad laboratories, Herts, UK). SYBR Green Supermix (Quanta Biosciences Gaithersburg, MD) was used with the following primer sequences (5′–3′) *CD109*—GAAGCTGTCCTCCTGTGACC; TCACAGCCAAAGTTCCATAAAG; *ENPP5*—TTGCACCCATGTCACAGAAT; CACTTATCACCTTCTTCTTCTCTCA *MIPOL1*—TGGTCATGCATGGAGTCTTG; ACTTACCTGCACCTCCCAGA *GAPDH*—CTGACTTCAACAGCGACA; TGCTGTAGCCAAATTCGTTG. The following programme was used: initial activation at 95°C for 10 min, followed by 40 cycles of denaturation at 95°C for 15 s, annealing at 59°C for 30 s and extension at 72°C for 30 s followed by fluorescence read. Primer efficiency was calculated and the expression of each gene in low and high vascularity tumours calculated relative to normal adult brain RNA (FirstChoice, Ambion) using the modified Pfaffl equation *R* = *E*_target_ ∆CT (CT target gene control−CT target gene sample)/*E*_control_ ∆CT (CT control gene control−CT control gene sample) with GAPDH as the control gene.

### Statistical analysis

Analysis was performed using SPSS 16 with comparison between groups undertaken using unpaired two-tailed *T* test. Survival analysis was performed using a Cox regression multivariate model and Kaplan–Meier plots (log-rank) for discrete groups. Array analysis was performed using Genespring software (Agilent, UK) with multiple unpaired *T* tests between groups and Benjamini–Hochberg multiple test correction applied throughout. *P* values of less than 0.05 were considered significant.

## Results

### Tumour cohort

The tumour cohort consisted of 150 pHGG with diagnosis confirmed by central pathological review, ranging in age (at diagnosis) from 2 days to 21 years, with a mean age of 7 years and 10 months. The cohort consisted of 88 males, 54 females and 8 cases where sex was not recorded, a male to female ratio of 1.6:1. Further details of the cohort have been previously published [41]. One hundred and thirty-six cases were obtained at first surgery and fourteen of the cases were obtained from surgery at first recurrence. Six of the children had survived previous (haematological) malignancies. Overall survival was also in line with other published series, with a 5-year-survival of 20% and median survival of 15 months from diagnosis. Survival tended to be extended with a diagnosis of AA compared to GBM (median 18 vs. 12 months, respectively, though *p* = 0.065).

### Immunohistochemistry

**Fig. 1:**
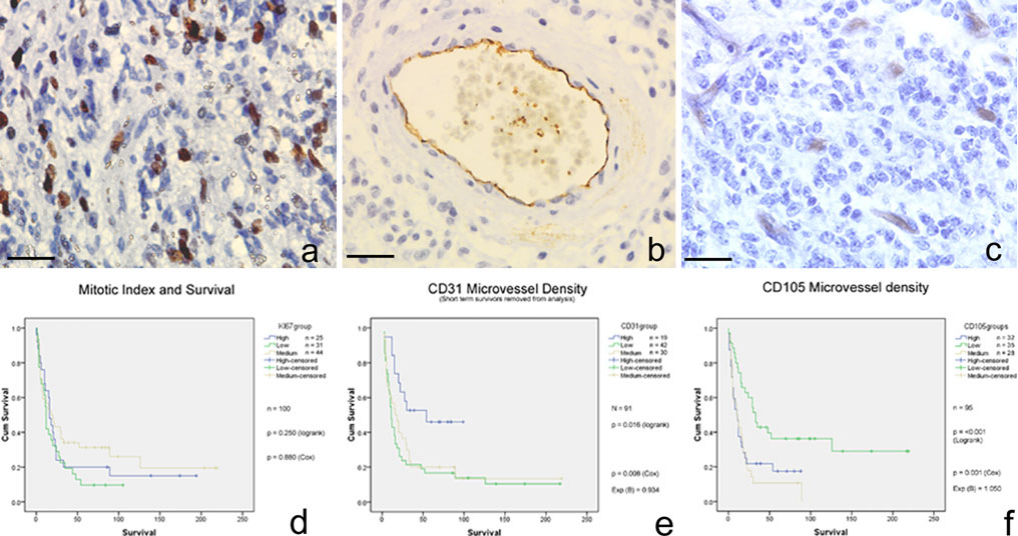
**a** Paediatric glioblastoma exhibiting typical level of Ki67 staining (brown nuclei positive for MiB-1). **b** Mature blood vessel in resected pHGG demonstrating positive endothelial staining for CD31 (brown lining of central lumen). **c** pHGG demonstrating high levels of immature small blood vessels staining positive for CD105 (brown structures). **d**, **e**, **f** Kaplan–Meier survival plots for Ki67, CD31 and CD105 levels, respectively (All *scale bars* 25 μm)

Ki67 staining data were acquired for 127 tumours with survival data available for 100 patients (Fig. 1). For individual tumour cores the proliferation index ranged from 0 to 52%. When averaged over multiple cores for each tumour, proliferation index ranged from 0 to 29.3% with a mean of 685 cells counted per tumour. Heterogeneity was seen within individual tumours, with some cores containing relatively high numbers of proliferating cells and other cores appearing relatively quiescent. No correlation could be demonstrated between overall proliferation index and length of overall survival (*p* = 0.880) (Fig. 1). When proliferation index was calculated specifically for cells in perivascular areas there was a significant correlation with overall patient survival (*p* = 0.012 Cox univariate) (see supplementary resource 4). Mitotic index across the wider tumour also had a significant correlation with survival on univariate analysis (*p* = 0.048).

CD31 immunohistochemistry data were available for 123 tumours, with survival data available for 97 patients. Mean number of positive staining vessels per core ranged from 0 to 69 vessels/core with an overall mean of 7.6 vessels/core. Mean number of vessels/core averaged over multiple cores for each individual tumour ranged from 0 to 48.5 vessels/core. For Kaplan–Meier purposes, tumours were split into groups of mean vessels/core less than 5, 5–10, and greater than 10, giving 55, 37 and 31 tumours in each group, respectively. This split was utilised as no previous studies have used objective criteria and it gave three roughly equal groups of samples.

There was no significant relationship between CD31 microvessel density (MVD CD31) and survival (*p* = 0.075 Cox regression). The data were then re-analysed with very short-term survivors removed from the analysis (patients who died less than 1-month post-surgery, *n* = 6) whose short survival may have been determined by surgical complications rather than tumour biology. There was now a significant relationship between MVD CD31 and survival (*p* = 0.008 hazard ratio = 0.934 Cox regression) with patients tending to survive longer if their tumour had a higher density of CD31 positive vessels (Fig. 1). There was no significant difference in levels of CD31 staining between GBM and AA (mean 7.1 vs. 8.6 vessels/core, respectively; *p* = 0.265 *T* test).

Immunohistochemical staining for CD105 was achieved for 118 tumours, with survival data available for 95 of these patients. Vessels staining positive for CD105 tended to be smaller and less mature in appearance (with higher levels of Ki67 positive cells and less pericyte/smooth muscle coverage) than vessels staining positive for CD31. It was also noticeable that some structures that histologically appeared as blood vessels did not stain positive for CD105. For individual tumours mean number of positively stained vessels ranged from 0 to 36 vessels/core, with single cores ranging from 0 to 59 vessels/core. Tumours were grouped into three levels of microvessel density for Kaplan–Meier analysis—those with less than 3 vessels/core (low), those with 3–8 (medium) and those with more than 8 vessels/core (high). As with CD31 IHC, no previous papers have used objective scoring systems, so the groupings are based on creating three groups of equal sample numbers.

**Fig. 2:**
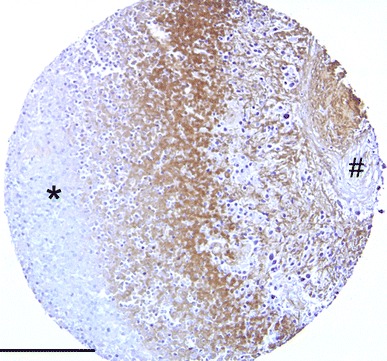
Immunohistochemistry for VEGF in the peri-ischaemic zone (positive staining brown) demonstrating viable peripheral tumour (*asterisk*) and central necrotic tumour core (*hash*) (*scale bar* 75 μm)

There was a highly significant relationship between microvessel density as defined by CD105 immunostaining (MVD CD105) and survival (Fig. 2). The greater the level of MVD CD105, the worse a patient’s survival was likely to be (*p* = 0.001 Cox regression, with hazard ratio of 1.05). All long-term survivors (>8 years), for whom data were available, were in the lowest group of MVD CD105 (<3 vessels/core). This relationship was identical on log rank testing by groups (*p* = <0.001) and if the short-term survivors were removed as for the CD31 analysis. The same relationship was also found using the individual peak core count for each tumour. There was a weak correlation between the peak CD31 and CD105 values for each tumour (*p* = 0.022 Spearman) but not the mean values. There was no relationship between mitotic index and microvessel density and the levels of CD105 staining were the same in GBM and AA (8.0 vessels/core and 6.4 vessels/core, respectively; *p* = 0.445 *T* test). There was a significantly shorter mean distance between areas of necrosis and CD105 positive vessels (121.9 μm) than between areas of necrosis and CD31 positive vessels (193.1 μm) (*p* = 0.001 *T* test).

VEGF immunohistochemistry data were available for 102 tumours, with survival data available for 79 of these patients. VEGF staining was variable with some cores being strongly positive and some completely negative. Even with a single core, expression could vary significantly, with VEGF positivity highest at the interface between necrotic and viable regions of cells (Fig. 2). 58 tumours had some degree of VEGF positivity. There was, however, no discernible relationship between extent of VEGF positivity and survival, age or any other variable (for survival, *p* = 0.818 Cox regression).

When multivariate analysis was performed for the 75 cases (Table 1) where information was available on all variables included in the model (age, sex, histological diagnosis, primary/recurrence, extent of surgery, administration of chemo/radiotherapy, mitotic index, MVD CD31, MVD CD105 and presence of VEGF) the significant variables affecting survival were MVD CD31 and MVD CD105 (*p* = 0.005 and <0.001, respectively), extent of surgery and administration of radiotherapy. The effect of MVD CD105 was slightly enhanced with hazard ratio increased to 1.084. The effect of MVD CD31 also achieved significance with an increased value correlating with improved survival chances (hazard ratio = 0.891). There was no significant association between age and any of the other factors studied.

**Table 1:** Multivariate Cox regression survival model for Paediatric High Grade Glioma

	*P* value	Exp (*B*)	95% Confidence interval for Exp (*B*)	
			Lower	Upper
Age	0.460	1.002	0.996	1.008
Sex	0.082	0.559	0.291	1.077
VEGF status	0.191	1.285	0.882	1.872
**MVD CD31**	**0.001**	**0.891**	**0.831**	**0.956**
**MVD CD105**	**<0.001**	**1.084**	**1.042**	**1.129**
Chemotherapy	0.461	0.766	0.377	1.556
**Radiotherapy**	**0.007**	**2.880**	**1.340**	**6.192**
**Resection status**	**0.001**			
**Gross total vs. biopsy**	**0.011**	**2.783**	**1.262**	**6.137**
Partial vs. biopsy	0.363	1.456	0.306	1.543

Variables significantly associated with survival in bold

### Gene expression

Gene expression analysis supervised on the basis of microvessel density was possible for 16 of the tissue microarray tumours that had been included in the gene expression cohort previously published [35]. The data from these 16 samples were uploaded into the Genespring analysis software. Quality control analysis for these samples was satisfactory with all quality control metrics passed. When multiple *T* testing with multiple test correction was performed, analysing the difference between samples on the basis of MVD CD31 no significant genetic differences were found. However, when the samples were analysed on the basis of MVD CD105, 13 genes were found to be significantly differentially expressed between samples in the low (<3 vessels/core) group and tumours in the medium and high groups (Table 2). Supervised hierarchical clustering was performed on the samples using the gene list with the results showing clear differences in expression profiles and the tumours of low MVD CD105 all clustering together in the block on the right of the cluster tree (online resource 2).

**Table 2:** Genes significantly differentially expressed between tumours with high and low CD105 microvessel density

Probe Set ID	Chromosome	Gene	Gene ID	Corrected *p* value	*P* value	Regulation (high to low vascularity)	FC absolute
203931_s_at	chr17q25.3	Mitochondrial ribosomal protein L12	MRPL12	0.0469	8.55E − 06	Down	1.34
205008_s_at	chr15q25.1	Calcium and integrin binding family member 2	CIB2	0.0469	1.42E − 05	Down	1.62
211086_x_at	chr4 q33	NIMA (never in mitosis gene a)-related kinase 1	NEK1	0.0469	7.15E − 06	Down	1.64
212787_at	chr14q24.3	YLP motif containing 1	YLPM1	0.0469	8.13E − 06	Down	1.34
215210_s_at	chr1p31.1	Dihydrolipoamide S-succinyltransferase	DLST	0.0469	1.71E − 05	Down	1.31
227803_at	chr6p12.3	Ectonucleotide pyrophosphatase/phosphodiesterase 5	ENPP5	0.0469	1.07E − 05	Down	9.25
229900_at	chr6 q13	CD109 molecule	CD109	0.0469	1.66E − 05	Down	1.91
233608_at	chr3p24.1	CDNA FLJ11929 fis, clone HEMBB1000434		0.0469	3.79E − 06	Up	2.31
235570_at	chr3 p24.1	CDNA FLJ36544 fis, clone TRACH2006378		0.0131	2.39E − 07	Up	2.75
240732_at	chr6q27	Transcribed locus		0.0469	1.57E − 05	Down	1.15
244246_at	chr14q21.1	Mirror-image polydactyly 1	MIPOL1	0.0274	1.00E − 06	Down	2.36
244836_at	chr6q24.2	Transcribed locus		0.0469	7.29E − 06	Up	1.21
1565877_at	chr6p22.3	Full length insert cDNA clone YP86C01		0.0469	1.15E − 05	Up	1.21

The genes that have been highlighted by this analysis include mirror image polydactyly-1 (*MIPOL1*), a candidate tumour suppressor gene and *ENPP5* (a closely-related molecule to *ENPP2* (autotaxin)). In view of their known tumour-related function these genes were selected for validation of the gene expression array data. On rtPCR significant differences in expression level relative to control (adult normal brain RNA) were confirmed for *MIPOL*-*1* and *ENPP5* (online resource 3).

### Immunofluorescence

**Fig. 3:**
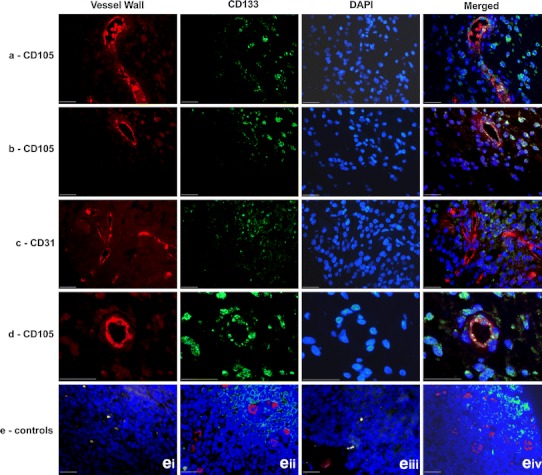
Co-immunofluorescence staining (blue nuclear DAPI staining): **a** CD105 (*red*) with CD133 (*green*) in an anaplastic astrocytoma, **b** CD105 (*red*) with CD133 (*green*) in a glioblastoma multiforme, **c** CD31 (*red*) with CD133 (*green*) in a glioblastoma multiforme, **d** CD105 and CD133 demonstrating co-staining of individual cells in vessel wall (*arrowed*) in a glioblastoma (*scale bars* all 25 μm). **e** Control sections of human tonsil demonstrating negative (*ei*) and positive (*eii*) controls for CD105 (*red*) and CD133 (*green*) and negative (*eiii*) and positive (*eiv*) controls for CD31 (*red*) and CD133 (*green*)

The proximity of CD133+ and CD133− cells to the closest CD31+ blood vessel was compared by immunofluorescence (Fig. 3) across 20 tumours, measuring 5,617 cells total. CD133+ cells were found to be significantly closer, on average, to CD31+ vessels than CD133− cells, by 5.83 (*p* < 0.001, 95% confidence interval 8.34–7.08 μm). 21 tumours were analysed from the co-staining of CD133 and CD105, with 9,525 cells measured. On average, CD133+ cells were also significantly closer than CD133− cells to CD105+ blood vessels, by 15.14 μm (*p* < 0.001, 95% confidence interval 14.89–16.07 μm), with the differences between CD133± cells greater for CD105 vessels than CD31 vessels (*p* = <0.001) (Table 3).

**Table 3:** Results comparing proximity of CD133± cells to CD31+ and CD105+ blood vessels and the difference between CD133± cells for each vessel type

		No. of cells	Mean distance from vessel (μm)		Significance of mean difference
			Mean	Mean difference	*P* value
CD31+ blood vessels	CD133+	2,326	41.04	5.83	<0.001
	CD133−	3,291	46.87		
CD105+ blood vessels	CD133+	4,387	40.81	−15.14	<0.001
	CD133−	5,138	55.95		

There was no significant difference between the mean distance from CD133+ cells to CD31 vessels or CD105 vessels (41.0 vs. 40.8 μm, respectively, *p* > 0.05 one way ANOVA for 4 samples, Tukey HSD post hoc test). CD133− cells were significantly further away from CD31+ vessels than CD105+ vessels. The basis for the increased differential for CD133± for CD105 vessels therefore seems to be an increased separation of CD133− cells from the blood vessel rather than increased proximity of CD133+ cells.

To investigate whether the proximity of CD133+ cells to CD105+ vessels was affected by the patient’s age, patients who were over 36-month-old at the time of diagnosis were compared with those 36 months or under. In the >36-month-group, CD133+ cells were on average, ~0.43 μm closer to CD105+ blood vessels than CD31+ vessels, however, the result was not statistically significant (*p* = 0.29). In contrast, CD133+ cells were significantly closer to CD31+ blood vessels in the 36 months or under group (by 4.73 μm, *p* < 0.001, 95% CI 5.56–3.90 μm). An increased proportion of CD133+ cells in an individual’s tumour showed a trend towards correlation with decreased survival but did not achieve statistical significance (*p* = 0.107 Cox regression).

To determine if the tumour type correlated with the distance of CD133+ cells from CD31+ and CD105+ blood vessels, measurements for patients with GBM and AA were analysed separately. The average proportion of CD133+ cells in GBM tumours (42.56%) was significantly greater than AA tumours (24.62%) (*p* = <0.001). Compared to AA, CD133+ cells in GBM were significantly closer to both CD31+ blood vessels (by 4.96 μm *p* < 0.001, 95% CI 6.95–2.97 μm) and CD105+ blood vessels (by 9.22 μm *p* < 0.001, 95% CI 11.62–6.82 μm). There was no significant association (p = 0.107) found between survival and proportion of CD133 expression though patient numbers were small.

The co-staining demonstrated cells that were positive for both CD133 (neural stem cell marker) and also for CD105 (endothelial marker) (Fig. 3). Up to 20% of vessel cells in some tumours demonstrated this co-staining.

## Discussion

CD105 has been suggested as a marker for angiogenic blood vessels in both CNS and other cancers [17]. Our study demonstrates a clear association between the number of blood vessels staining positive for CD105 (a subset of the total number of vessels) and the overall survival of patients with pHGG. This association is present despite the variety of treatments these patients underwent (biopsy versus resective surgery; radiotherapy; various forms of chemotherapy).

There are several possible explanations for the link between CD105 and survival. When a tumour outgrows its blood supply, ischaemia and necrosis result, leading to generation of angiogenic mediators and the recruitment of new blood vessels and epithelial precursor cells. This new blood vessel network may then fuel further tumour growth by supplying increased quantities of oxygen and other necessary metabolites, leading to brain invasion and hence worsened prognosis.

Many of the tumour-specific new vessels are small and highly abnormal in morphology. The lumen is often small such that the amount of blood carried by these vessels is minimal, although there can be many vessels. A second explanation for the association between the number of novel vessels and tumour behaviour is that the vessel architecture provides a microenvironment that is supportive of a subset of tumour cells (putative stem-like cells) that provide resistance to radiotherapy or chemotherapy [8]. This stem-like population is known to favour being in close association with vessels and it is recognised that tumour xenografts grow faster if co-grafted with endothelial cells [49]. The co-existence of endothelial cells within a tumour may provide a favourable scaffold to which the glioma cells can attach, facilitating tumour growth via more favourable cell–cell interactions.

A third possible mechanism is also linked to the cancer stem cell hypothesis. CD105 is well recognised as a marker for mesenchymal stem cells [9]. Some high grade gliomas demonstrate mesenchymal features with a subset of glioblastoma featuring frank sarcomatous de-differentiation as seen in gliosarcoma [31]. Some of the structures staining positive for CD105 could potentially be mesenchymal stem-like cells invading the brain parenchyma in response to chemotactic cues from the tumour and hypoxia. Recruitment of stem-like cells could lead to increased vessel growth, paracrine stimulation of tumour growth or further creation of a favourable microenvironment. It may even be that aggressive HGG de-differentiate to the point where they can re-acquire mesenchymal characteristics that are not normally displayed on central nervous system cells. Recent work has demonstrated the existence of this phenomenon in adult HGG (termed vasculogenic mimicry) [39, 42, 45]; our work provides evidence that this phenomenon may also exist in pHGG.

There was a weak positive correlation between MVD CD31 and overall survival once very short-term survivors were removed from the analysis. This could be explained by inclusion of normal brain parenchymal vessels into the tumour and thus into the surgical specimen. In this case the vessels could be a sign that the tumour is still at an early stage of spread through the brain and that normal tissue continues to survive within the tumour. At a later stage of tumour growth the ischaemic conditions generated by the cancer would lead to death of the normal tissue and selection for hypoxia-resistant tumour cells, along with tumour-induced CD105 positive blood vessels.

An alternative explanation is that the vessels staining positive for CD31 are indeed formed as part of tumour growth and are not simply bystander vessels incorporated into the tumour as it invade surrounding brain. In this case the slight survival benefit may be explained due to the level of vessel maturity. Tumours that are more differentiated may in turn produce vessels that are better differentiated and will thus likely display higher levels of the mature vessel wall marker CD31. Tumours with higher levels of mature cells and less anaplasia will be more likely to have a better prognosis as they may grow more slowly and invade less extensively into surrounding brain. Alternatively, CD31 positive vessels may be more efficient in oxygenating the tumour parenchyma due to their greater maturity and increased diameter. The improved tissue oxygenation would then result in a decreased drive for tumour cells to migrate out of hypoxic areas and invade surrounding brain. Improved tumour oxygenation could also potentially have other effects such as fuelling higher tumour cell metabolism and turnover, or by influencing the level of stem-like behaviour shown by tumour cells.

The lack of significant correlation between clinical factors, MiB-1 and VEGF may also have various explanations. One might expect mitotic index to correlate well with prognosis as faster growing tumours should logically lead to shorter survival, and indeed this has been found to be the case in some CNS tumour types [44]. It is clear that in paediatric HGG in this study, expression of MiB-1 is highly variable across individual tumours. Certain patches of cells within an otherwise bland field may have a high mitotic rate, but the overall average across the count may be low.

It may be that the sampling carried out in this study was simply not extensive enough to accurately gauge the true proliferation rate for each tumour and negative Ki67 staining may not necessarily imply quiescence, just that negative cells have not been stained at the particular phase of mitosis when the target protein is present. There is also an issue with the scoring system as to how one incorporates a small area of highly active tissue which may be the most prognostically significant area into the overall assessment of a tumour which may have large areas of less proliferative tissue. By contrast, scoring proliferation by counting mitotic figures across multiple high powered fields showed a significant relationship with survival, suggesting that rate of cellular proliferation has an important influence on the behaviour of these tumours. The interesting finding that proliferation index in the perivascular population is significantly correlated with survival supports the idea that it is the proliferation rate of sub-populations within the tumour rather than the total cell proliferation that is critical. Tumours with increased perivascular proliferation may have the capacity to increase their nutrient supply more quickly than tumours with slower rates of blood vessel growth. This increased proliferation could also be generating favourable microvascular niche environments at a faster rate, thus enhancing tumour growth and possibly promoting the stem-like fraction of cancer cells. Indeed, it may even be that the proliferative cells identified in the vessel walls are tumour cells undergoing a vasculogenic mimicry type process. It is worth noting that proliferation index is not currently part of the WHO grading criteria for astrocytomas because of the difficulties in obtaining a consistent inter-observer value for individual tumours.

VEGF is also locally and specifically expressed at the hypoxic/normoxic interface where ischaemic conditions trigger the angiogenic switch. Even within a single core, a band of expression could often be seen with low levels on either side of VEGF staining in the necrotic core and in the viable periphery. It again seems that results for VEGF are likely to be highly dependent on the precise location within the tumour from where the sample came. In some tumours this region of maximal expression will not be visualised as the specimen cores will have been extracted from other regions of the tumour. Equally, on molecular biological studies, only a small part of the tumour is analysed and molecular differences may well exist within different regions of the same tumour as well as between different patients’ tumours.

The list of genes showing varying levels of expression between tumours with high and low microvessel density as measured by CD105 immunohistochemistry shows several candidate genes of potential interest. Mirror image polydactyly 1 (*MIPOL1*) is located on 14q and was initially identified in a patient with this deformity [26], although the mechanism of its action has not been identified. It is known to be active in CNS development as loss of this gene can also give rise to craniofacial defects and agenesis of the corpus callosum [25]. *MIPOL1* was implicated as a putative tumour suppressor gene in nasopharyngeal carcinoma (NPC), where chromosome 14 loss (including the *MIPOL1* locus) is common [12]. Re-expression of *MIPOL1* in NPC cell lines greatly reduced their ability to proliferate or form tumours in xenograft experiments. In our study *MIPOL1* was found to be 2.4-fold downregulated in the higher vascularity tumours. MIPOL1 protein is located in the nucleus and seems to exert its effects by up regulating expression of *p27* (*WAF1/CIP1*) and *p21* (*KIP1*), which are also implicated as angiogenic markers in other malignancies.

Another gene of interest highlighted by this analysis is *CD109* (chromosome 6), a cell surface antigen which was initially studied in CD34 positive acute myeloid leukaemia cell lines, but has subsequently been found to be expressed on a variety of haematopoietic cells and endothelial structures [33]. It has also been implicated as a marker for a subset of mesenchymal stem cells. Its function seems to be negatively regulate TGFβ signalling by direct action on the receptor. Post-translational processing of the molecule into smaller fragments seems to be necessary for the utilisation of the TGFβ signalling pathway by neoplastic cells [20]. It has been implicated in breast cancer [23] (where RNAi knockdown of *CD109* suppressed malignant growth and CD109 shed from the cell surface has a pro-growth function), squamous cell carcinoma of the oral cavity [21] and is upregulated in various tumour cell lines including GBM cell lines [22]. In our project *CD109* was found to have a 1.9× fold change between high and low vascularity tumours.

*ENPP5* was highly differentially expressed (9.2× fold change between groups). This molecule has not been widely studied but the closely-related molecule ENPP2 (autotaxin) has been widely implicated in neoplasia and is the focus of considerable efforts to develop small molecule inhibitors [30]. It produces lysophosphatidic acid, a known stimulant for cell proliferation, survival and invasive behaviour. Other genes implicated in our study include *MRPL12*, a downstream target of the *REIC/Dkk3* tumour suppressor in prostate cancer [11], *CIB2* (a potential stress response protein linking to apoptotic pathways) [48], *NEK1* (down regulation of which may worsen the ability of the cell to repair DNA damage) [36] and *DLST* (a component of the respiratory chain). These genes will be further investigated in novel three dimensional cell culture models of angiogenesis we are currently developing.

This study also investigated whether cells marked with the putative stem cell marker, CD133, were found in closer proximity to the glioma vasculature than CD133− cells. The results indicated that CD133+ cells were found significantly closer to both CD105+ and CD31+ blood vessels than CD133− cells; this result is consistent with other studies which have shown that glioma cells expressing other stem cell markers, such as Nestin, were located closer to brain tumour vessels than Nestin negative cells [8, 13, 46]. The relatively high rates of CD133 positivity reported in our study may be related to the elevated levels of stem-like cells found in the developing brain compared to the adult brain. We also specifically selected the peri-vascular areas as the focus for this study rather than examining the tumour as a whole. Several groups have reported that the peri-vascular niche is an area enriched for stem-like cells [8] and by focusing on this area, one might expect our rates of CD133 positivity to be higher than other groups which assess the rate of positivity across the tumour in its entirety.

It was observed that the difference of the proximity of CD133+ cells/CD133− cells to CD105+ vessels was greater than that to CD31+ blood vessels. Furthermore, when comparing GBM and AA tumour samples, CD133+ cells were found significantly closer to CD105+ blood vessels in GBM than in AA, but this difference between GBM and AA was much less for CD31+ blood vessels. This suggests that CD105+ vessels may have a more important role than CD31+ vessels in the vascular microenvironment for stem-like cells, especially in grade IV tumours. This could be related to the presence of necrosis in the grade IV tumours and the greater proximity of CD105 positive vessels to necrosis (compared to CD31 positive vessels) suggesting a role for CD105 in hypoxic areas.

An interesting finding was that the average proportion of total cells counted that were CD133+ cells in GBM which was greater than in AA. This may be associated with the higher histological grading of GBM, thus supporting studies that suggest CD133 expression can be used as a prognostic factor [3] though CD133 did not achieve prognostic significance in our study. It is possible that not all of the CD133+ cells counted will have been glioma CSC’s with positive staining also depending upon the oxygen concentration and expression of the glycosylated AC133 epitope at different stages of the cell cycle [5, 24, 32].

Some CD31 and CD105 endothelial cells co-expressed CD133, as part of the vessel wall or as single cells. CSC’s in GBM tumours are suggested to be able to produce differentiated neoplastic endothelial cells, forming part of the hyperplastic GBM vasculature [39]. Our work suggests that this phenomenon may also exist in paediatric HGG and contributes to the development of tumour-associated vasculature in children.

This study demonstrates that CD105 positive vessels have a significant role in the biology of pHGG and exert an influence over prognosis even in a heterogeneously treated cohort. Expression of different levels of CD105 by tumours is associated with wider changes in gene expression, allowing the identification of novel genes not previously implicated in CNS tumour angiogenesis. One possible explanation for the prognostic influence of CD105 is through its role in developing the microvascular niche in which stem-like cancer cells preferentially inhabit. Our data provide evidence that this supportive micro-environment may be preferentially found in the proximity of CD105 positive vessels as opposed to blood vessels stained by other endothelial markers. This suggests that CD105 or other components of the TGF beta pathways may be potential therapeutic targets for the treatment of pHGG. Future studies of the HGG microvascular niche should focus on the environs of CD105+ vessels and their interplay with, and possible development from, CD133+ tumour cells.

## Electronic supplementary material

Below is the link to the electronic supplementary material.

Supplementary material 1 (TIFF 17945 kb)

**Online resource 1** Central blood vessel stained positively for CD105 (red) with illustration of measurement technique (multiple lines) to nuclei of surrounding cells (DAPI blue stained)(scale bar 20 μm)

Supplementary material 2 (TIFF 4068 kb)

**Online resource 2** Supervised hierarchical clustering of 16 pHGG demonstrating separation of high and low MVD CD105 tumours based on expression of differentially expressed genes

Supplementary material 3 (TIFF 6156 kb)

**Online resource 3** Validation by realtime polymerase chain reaction for three selected genes – ENPP5, CD109, MIPOL-1 with ENPP5 and MIPOL1 significantly differentially expressed

Supplementary material 4 (TIFF 1101 kb)

**Online resource 4** Kaplan–Meier plot stratified by level of vessel wall cell Ki67 positivity, demonstrating reduced overall survival in tumours with high levels of vessel wall cell proliferation.
