# Supplementary figures and images for: CD105 (Endoglin) exerts prognostic effects via its role in the microvascular niche of paediatric high grade glioma
Source: Acta Neuropathol. 2012 Feb 7;124(1):99–110. doi: 10.1007/s00401-012-0952-1 (PMC3377898; doi:10.1007/s00401-012-0952-1)

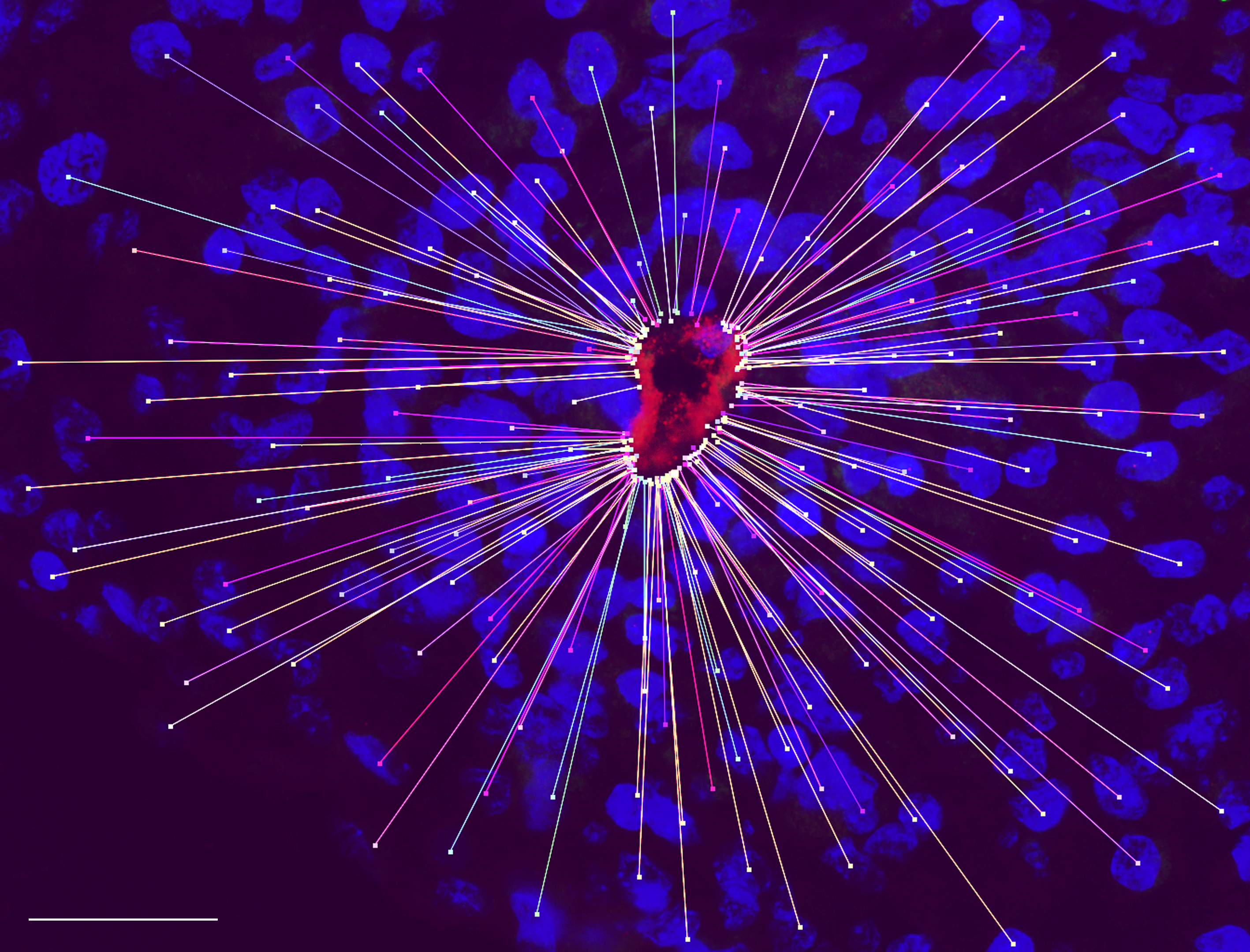

Supplement: Supplementary file 1 — Supplementary material 1 (TIFF 17945 kb) [file 401_2012_952_MOESM1_ESM.tif]

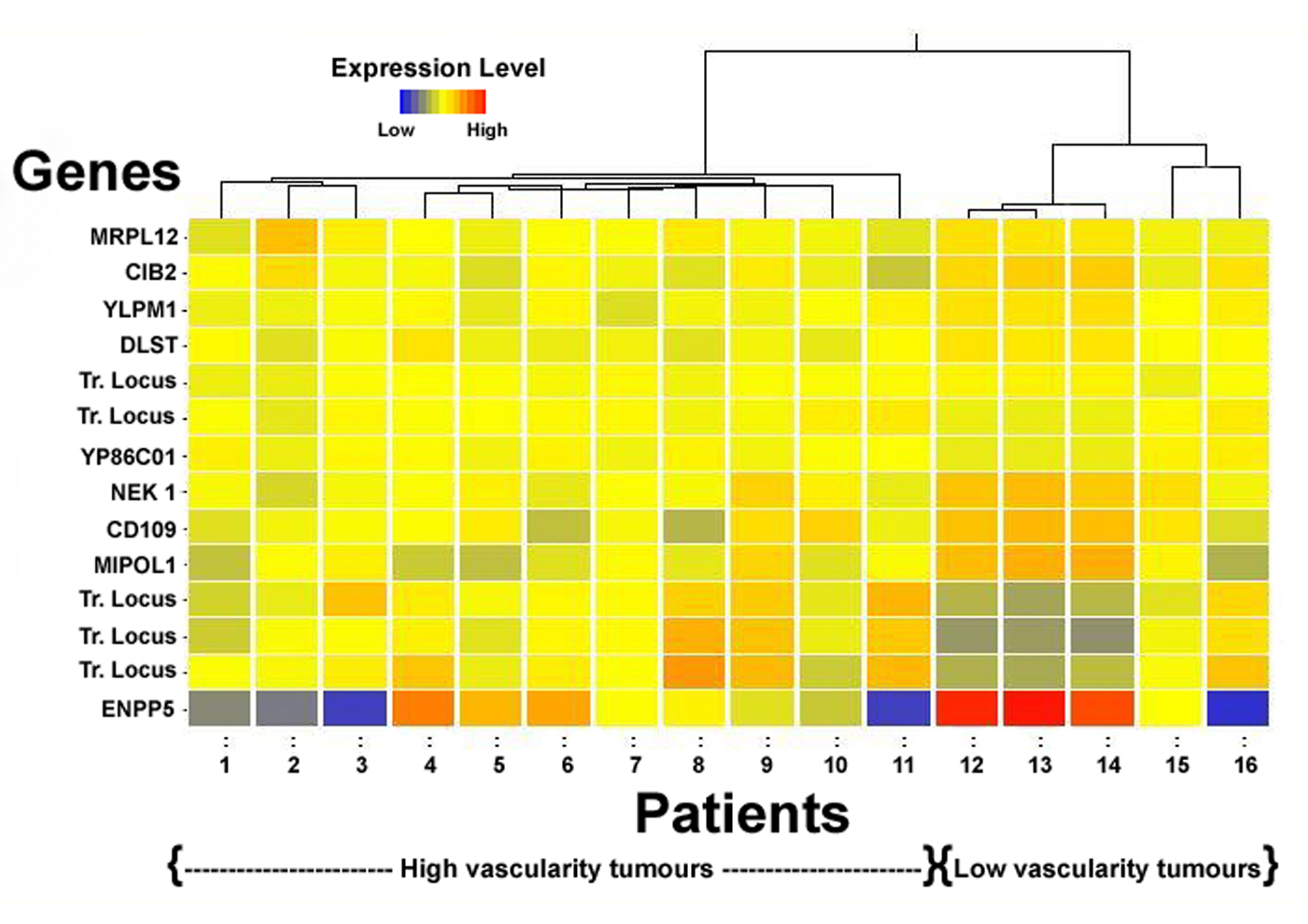

Supplement: Supplementary file 2 — Supplementary material 2 (TIFF 4068 kb) [file 401_2012_952_MOESM2_ESM.tif]

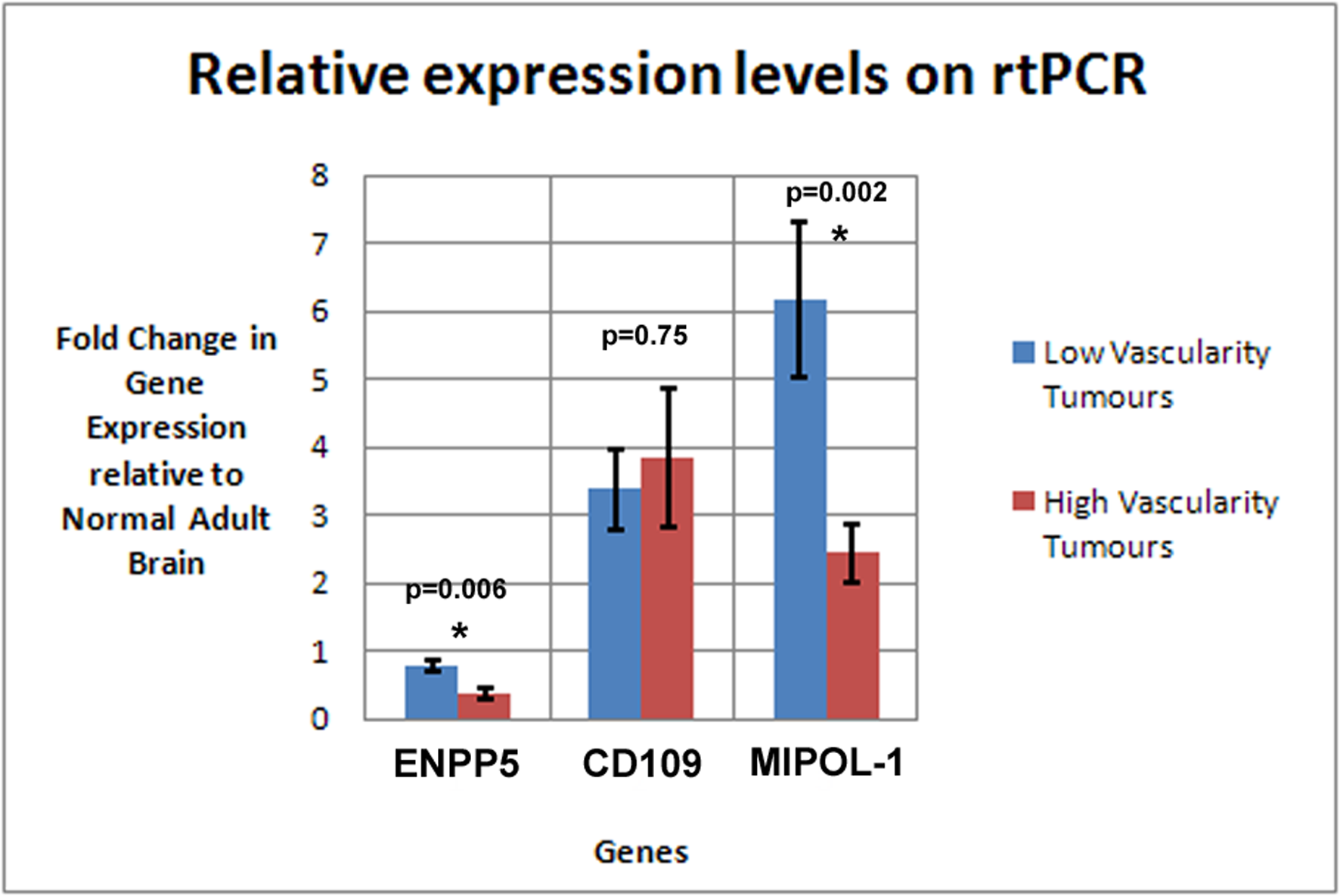

Supplement: Supplementary file 3 — Supplementary material 3 (TIFF 6156 kb) [file 401_2012_952_MOESM3_ESM.tif]

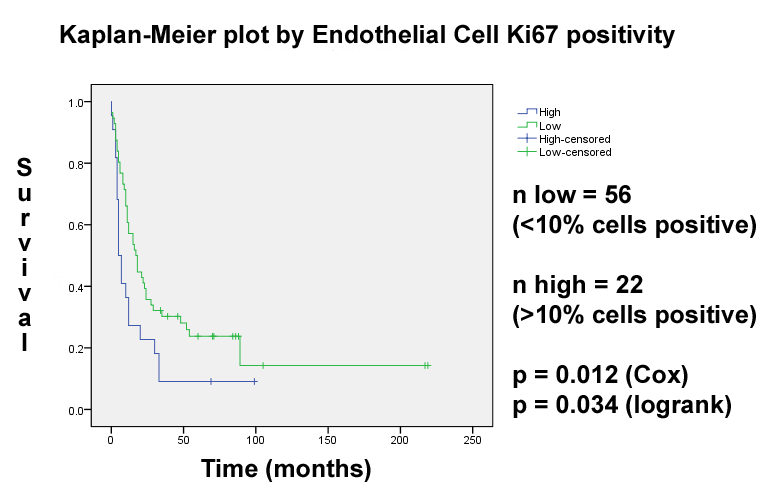

Supplement: Supplementary file 4 — Supplementary material 4 (TIFF 1101 kb) [file 401_2012_952_MOESM4_ESM.tif]
